# Development and Application of Anthocyanin-Based Complex Polysaccharide Gels Based on Blueberry Pomace for Monitoring Beef Freshness

**DOI:** 10.3390/gels11060385

**Published:** 2025-05-23

**Authors:** Jingxi Zhi, Fuqian Xu, Shuhuan Yu, Jiahui Hao, Jie Wang, Ziluan Fan

**Affiliations:** 1 College of Life Science, Northeast Forestry University, 26 Hexing Road, Xiangfang District, Harbin 150040, China; aulin-zjx@nefu.edu.cn (J.Z.); xufuqian@nefu.edu.cn (F.X.); yushuhuan@nefu.edu.cn (S.Y.); haojiahui5832@163.com (J.H.); wj18365936610@163.com (J.W.); 2 Hangzhou Institute for Advanced Study, School of Life and Health Sciences, University of Chinese Academy of Sciences, 1 Xiangshan Branch Lane, Xihu District, Hangzhou 310024, China; 3 Key Laboratory of Forest Food Resources Utilization, Harbin 150040, China

**Keywords:** blueberry pomace anthocyanins, complex polysaccharide gels, freshness monitoring, chilled beef

## Abstract

This study aimed to develop a green and sustainable composite polysaccharide gel with antioxidant activity and freshness-monitoring properties. Blueberry pomace was repurposed to extract anthocyanins (BA), which were incorporated into chitosan (CS)/polyvinyl alcohol (PVA) and starch (S)/PVA matrices to prepare pH-indicating composite polysaccharide gels. The anthocyanin solution exhibited significant colorimetric responses to pH 2–14 buffer solutions. Comparative analyses revealed distinct performance characteristics: the CS/PVA-BA gel showed optimal elongation at break, low hydration (8.33 ± 0.57% water content), and potent antioxidant activity (DPPH: 73.59 ± 0.1%; ABTS: 77.47 ± 0.1%), whereas the S/PVA-BA gel demonstrated superior tensile strength and pH-responsive sensitivity. Structural characterization via FT-IR and SEM confirmed molecular compatibility between BA and polymeric matrices, with anthocyanins enhancing intermolecular hydrogen bonding. Applied to chilled beef (4 °C) freshness monitoring, the CS/PVA-BA gel exhibited color transformations from magenta-red (initial spoilage at 48 h: TVB-N > 15 mg/100 g, TVC > 4.0 lg CFU/g) to bluish-gray (advanced spoilage by day 6), correlating with proteolytic degradation metrics. These findings established a multifunctional platform for real-time food quality assessment through anthocyanin-mediated color changes in the composite gels, coupled with preservation activity, highlighting their significant potential as intelligent active packaging in the food industry.

## 1. Introduction

With the improvement of living standards, the market demand for chilled beef has significantly increased. Beef, rich in protein, vitamins, and minerals, is an essential component of the human diet [1]. However, due to microbial contamination, lipid oxidation, and protein degradation, chilled beef is highly susceptible to spoilage, compromising its quality and commercial value [2,3]. Currently, consumers primarily rely on expiration dates and appearance to assess freshness, which lacks scientific rigor [4]. Therefore, food packaging technology requires further development, and there is an urgent need to create intuitive and rapid methods for monitoring food freshness.

In recent years, intelligent packaging has emerged as a novel solution, particularly in systems that enable the visual judgment of food quality through color changes. pH-sensitive substances blended with polymers can form packaging gels that display distinct colors under varying pH conditions, achieving the real-time monitoring of food freshness [5,6]. However, indicators are categorized into synthetic and natural pigments. Synthetic indicators, such as methyl red, bromocresol green, and bromocresol purple [7], pose safety risks due to potential toxicity [8]. In contrast, natural pigments like anthocyanins have gained prominence. Anthocyanins, as polyphenolic flavonoids, constitute a class of water-soluble natural pigments widely present in plants [9]. Anthocyanins, characterized by their ease of extraction and pH-responsive color changes, were extracted and purified from blueberry pomace in this study to serve as indicators in multifunctional gels. These compounds also exhibit potent antioxidant activity, enabling their utilization in active packaging systems to extend the shelf lives of food products [10]. Blueberry pomace, a processing byproduct typically discarded or used as low-value fertilizer, poses environmental risks when landfilled or incinerated. Direct disposal generates greenhouse gases and leachate, which contaminate soil and water systems [11]. However, blueberry pomace retains significant amounts of underutilized anthocyanins. Utilizing this low-cost byproduct drastically reduces the production costs of anthocyanins. Extracting anthocyanins from blueberry pomace not only achieves a “waste-to-resource” transformation but also aligns with sustainable development goals. Furthermore, compared to synthetic pigments, anthocyanins exhibit higher biological activity and lower costs.

To ensure food safety, freshness-indicating gels are typically fabricated from biodegradable, biocompatible, and non-toxic materials such as starch, chitosan, carboxymethyl cellulose (CMC), and their composites [12]. Natural renewable resources, including polysaccharide-based (e.g., cellulose, starch, and chitosan) and protein-based materials (e.g., corn protein, soy protein isolate, and gelatin), are increasingly utilized due to their low costs and abundant availability [13]. Chitosan, a natural polysaccharide extracted from crustacean shells, is characterized by biodegradability, antibacterial properties, and film-forming ability [14]. Under acidic conditions, the amino groups (−NH_2_) in chitosan molecules undergo electrostatic interactions with negatively charged molecules to form gels [15]. In food packaging applications, chitosan’s antibacterial properties extend the shelf lives of products while its film-forming ability enables the formation of uniform film structures. Starch, a widely available and low-cost natural polymer, exhibits excellent film-forming properties and biodegradability. When starch particles are gelatinized by heating, amylose and amylopectin recombine through hydrogen bonds to form a three-dimensional network structure [16]. This network binds water molecules via hydrogen bonds, conferring viscoelasticity and moisture retention to the gel. For example, Zong et al. [17] used purple potato anthocyanins as indicators to prepare complex polysaccharide gels with starch and gelatin and applied them to the freshness monitoring of the Flammulina velutipes mushroom. Feng [18] fabricated corn starch (CS)/carboxymethyl cellulose (CMC) composite gels with blueberry pomace anthocyanins (BA) as indicators, demonstrating an effective monitoring of beef freshness. These studies validated the feasibility of anthocyanins as functional indicators. Polyvinyl alcohol (PVA), a synthetic water-soluble polymer, offers excellent film-forming ability, mechanical strength, and chemical stability. It exhibits good compatibility with natural components (e.g., anthocyanins and essential oils) and can stably incorporate functional additives [19]. However, pure starch films may suffer from limited mechanical strength or poor water resistance while the biodegradability of PVA requires further enhancement. Blending chitosan with PVA improves the mechanical strength of gels and imparts antimicrobial properties [20]. Barbara Merz et al. [21] investigated chitosan/polyvinyl alcohol (PVA) gels incorporating anthocyanins, analyzing their thickness, microstructure, moisture content, and hydrophobicity, and applied them to monitor shrimp freshness. When starch is mixed with polyvinyl alcohol, its brittleness can be improved. Chen et al. [22] immobilized con go red (CR) and anthocyanins (ATH) in starch/PVA/glycerol gels, highlighting the excellent gel-forming properties of polysaccharide-PVA blends.

This study extracted anthocyanins from blueberry pomace and prepared smart indicator films by combining starch and chitosan with polyvinyl alcohol (PVA) as film-forming matrices, using glycerol as a plasticizer. The films were applied to monitor the freshness of chilled beef, providing a theoretical foundation for the use of smart packaging in meat freshness detection. The resulting gel demonstrated significant potential for application in active food packaging and real-time freshness indication, advancing sustainability goals in the food industry.

## 2. Results and Discussion

### 2.1. Quantification and Characterization of Anthocyanins from Blueberry Pomace

**Figure 1:**
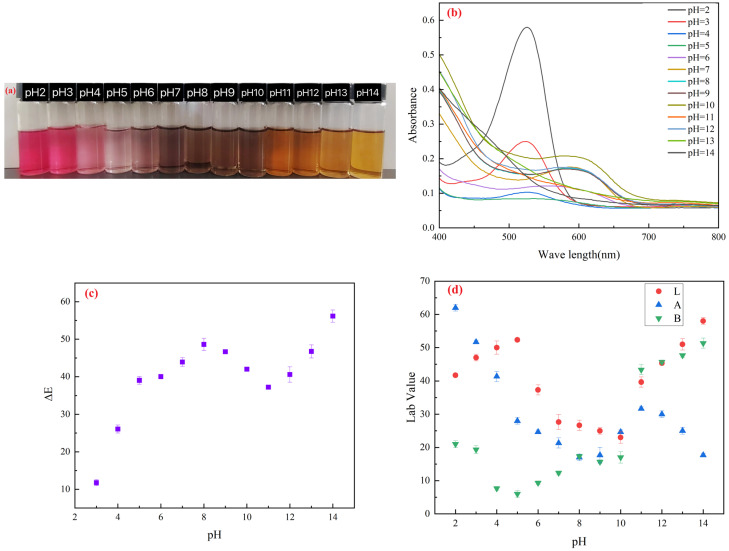
(**a**) Color changes in blueberry pomace anthocyanin solution at pH 2–14; (**b**) UV-VIS spectrum of blueberry pomace anthocyanin solution at pH 2–14; (**c**) ΔE values and (**d**) lab values of blueberry pomace anthocyanin solution at pH 2–14. Vertical bars represent the standard deviations of three replicates.

The total anthocyanin content extracted from blueberry pomace was 29.31 ± 1.18 mg/g. The anthocyanin solution exhibited distinct color changes under varying pH conditions. As shown in Figure 1a, the solution appeared red at pH 2–3, transitioned to pink at pH 4–6 with gradual fading, turned purple at pH 7–8, shifted to blue and green in alkaline conditions (pH 9–11), and finally turned yellowish-brown at pH 12–14 [23]. The color of blueberry pomace anthocyanins varied significantly in different pH ranges. Figure 1b displays the UV-Vis spectra of blueberry pomace anthocyanins. In acidic solutions (pH 2–5), the maximum absorption peak appeared near 523 nm. As the pH increased, the red peak shifted to 540 nm with reduced absorbance. At pH 8, the absorption maximum shifted to 576 nm, while under alkaline conditions (pH 10–14), it stabilized around 600 nm. These spectral shifts corroborated the structural transitions observed in color changes.

This color variation and shift in maximum absorption wavelength arose from the highly conjugated system within anthocyanin molecules, which underwent structural transformations in response to pH changes. Under strongly acidic conditions (pH < 3), anthocyanins primarily exist as red-colored flavylium cations in solution, exhibiting lower maximum absorption wavelengths [24]. As the pH increases, the flavylium cations undergo hydration to form colorless carbinol pseudobases, resulting in diminished red coloration and reduced absorbance [25]. At neutral to slightly alkaline conditions (pH 7–9), anthocyanins adopt a blue quinonoidal base structure. When the pH exceeds 9, further deprotonation leads to the formation of yellowish-brown chalcones [26,27].

The colorimetric analysis data of anthocyanin solutions under different pH conditions are shown in Figure 1c,d. The L value (lightness) of the solution increased under acidic conditions, decreased under neutral conditions (resulting in darker solutions), and gradually rose again as the pH increased to alkaline levels, restoring the solution’s brightness. With increasing pH, the a value (redness) gradually decreased, reaching its lowest level at pH 10. Subsequently, as the alkalinity further increased, the a value initially rose and then declined again. This trend aligned with the visual observations shown in Figure 1a, where the redness diminished as the pH increased from 2 to approximately 7. The b value (yellowness) increased under alkaline conditions, accompanied by a color transition of the solution from green to yellowish-brown. ΔE (Delta E) is a metric used to quantify color differences, representing the numerical value of perceptual differences between two colors in a uniform color space. When the ΔE value exceeds 5, the color variation becomes visually perceptible [28]. Since all ΔE values of the indicator were greater than 5, the color changes in the indicator could be distinctly differentiated by the naked eye.

### 2.2. Characterization and Analysis of Complex Polysaccharide Gels

#### 2.2.1. SEM Analysis of Gels

**Figure 2:**
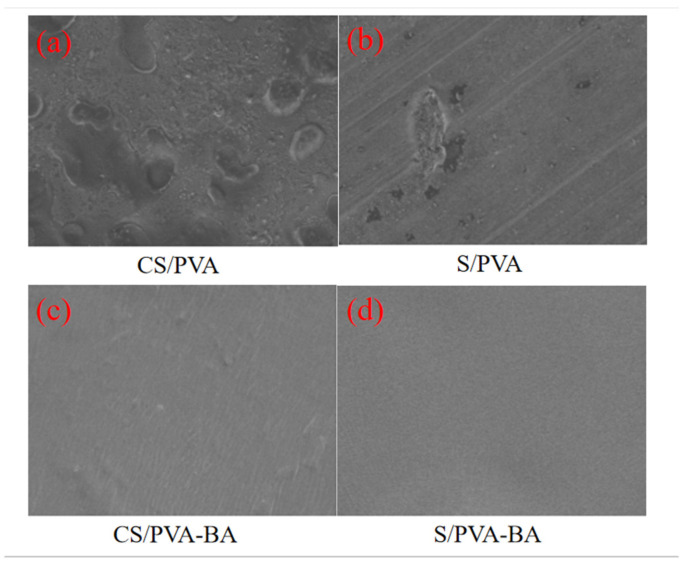
Scanning electron microscopy images (1000×) of surface of (**a**) CS/PVA, (**b**) S/PVA, (**c**) CS/PVA-BA, and (**d**) S/PVA-BA gel.

The dispersion state and compatibility of components within a composite gel can be determined through SEM (scanning electron microscopy) images. The surface scanning electron microscopy images of the four gels are shown in Figure 2. Typically, a smooth and flat surface with a uniform structure, free of protrusions or wrinkles, indicates excellent film-forming properties and high compatibility among the gel matrix components [29]. On the contrary, there were obvious protrusions and pores, indicating that the gel surface was rough. Figure 2 shows the surface microstructure of the four gels magnified 1000 times. Compared with the gels without blueberry pomace anthocyanins, the CS/PVA-BA gels and S/PVA-BA gels with blueberry pomace anthocyanins added were tighter and smoother with no obvious bumps and folds. This suggests that blueberry anthocyanins (BA) not only exhibit good compatibility with chitosan (CS), polyvinyl alcohol (PVA), and starch (S) but also enhance intermolecular interactions between CS-PVA and S-PVA matrices [30]. The anthocyanins effectively bind to polysaccharides and disperse uniformly within the film, which aligns with the findings reported by Merz et al. [21].

#### 2.2.2. Infrared Spectrum Analysis of Gel

**Figure 3:**
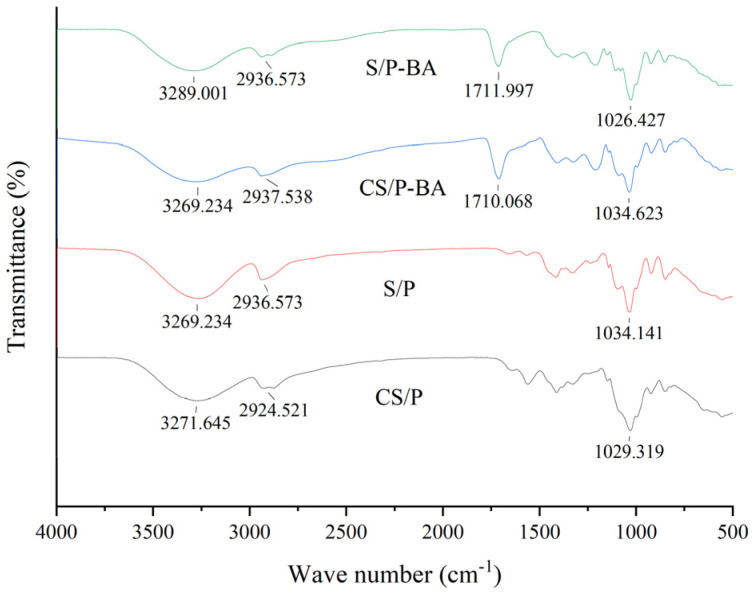
Fourier-transform infrared (FT-IR) spectrum analysis of CS/PVA, S/PVA, CS/PVA-BA, and S/PVA-BA gel.

FT-IR can be used for the qualitative analysis of substances and effectively characterizes the molecular structure and cross-linking of organic compounds. It can be seen in Figure 3 that the absorption peak at 3289–3300 cm^−1^, indicating that the gel was in the range of 3200–3600 cm⁻^1^, belonged to the stretching vibration of free amino (−NH_2_) in the chitosan (CS) and hydroxyl (−OH) of polymers (such as PVA and starch) [31]. Aliphatic C-H vibrations at 2937–2986 cm^−1^ are related to hydrophobic groups in the plasticizer or polymer side chain, which may regulate the gas permeability of a gel [32]. The characteristic front at 1710 cm^−1^ is caused by the stretching vibration of C=C on the aromatic ring skeleton in anthocyanins. In addition, the C-O vibration peak at 1034–1094 cm^−1^ indicates the hydrogen bond between the polysaccharide matrix (CS and starch) and the plasticizer [33], and the low frequency shift of CS/P at 1029 cm^−1^ reflects the strengthening effect of chitosan molecular chain rigidity on the hydrogen bond network [34]. Therefore, it can be seen from the FT-IR spectrum that the structure of the material was mainly affected by the intermolecular force, and the chemical composition of the gel-forming substrate did not change.

#### 2.2.3. Analysis of Physical Properties of Gel

It can be seen from Table 1 that there were differences in the thickness of the four gels. In the process of gel forming preparation, the molecular cohesion between gel-forming substrates will lead to differences in gel thickness [35]. The addition of anthocyanins also increased the thickness of the gel.

Tensile strength and elongation at break are fundamental indicators for evaluating the performance of packaging materials. Superior mechanical properties enhance resistance to compression during food transportation and improve product protection. Significant differences (*p* < 0.05) were observed in tensile strength and elongation at break among the four indicator films. Chitosan (CS), with its flexible molecular chains, exhibits high elongation at break. However, the incorporation of anthocyanins disrupts the crystalline structure of the film matrix, reducing rigidity. The hydrogen bonding between CS amino groups (−NH_2_) and polyvinyl alcohol (PVA) hydroxyl groups (−OH) may be insufficient in cross-linking density, leading to molecular chain slippage and decreased tensile strength [36]. Starch–PVA films exhibit poor compatibility, resulting in high brittleness. While these films demonstrate higher tensile strength, their elongation at break is significantly lower than that of chitosan-based films [37,38]. The addition of anthocyanins enhances intermolecular interactions, improves structural stability, and partially enhances mechanical properties [39]. Chitosan and PVA films form strong intermolecular interactions, limiting water molecule penetration and maintaining a low water content [40]. The incorporation of anthocyanins further reduces gel hydration due to interactions between chitosan/starch and anthocyanin molecules, which restrict the availability of polysaccharide hydroxyl groups and weaken the water binding capacity.

Water vapor permeability (WVP) is a critical parameter for assessing the moisture barrier performance of food packaging materials. A lower WVP indicates superior resistance to external moisture ingress [41]. As shown in the table, chitosan–PVA gels exhibit lower WVP compared to starch-based gels. However, the addition of anthocyanins increases WVP, likely attributed to the hydrophilic phenolic hydroxyl groups in blueberry anthocyanins (BA), which enhance the film’s water adsorption capability [42].

### 2.3. Antioxidant Properties of the Complex Polysaccharide Gels

**Figure 4:**
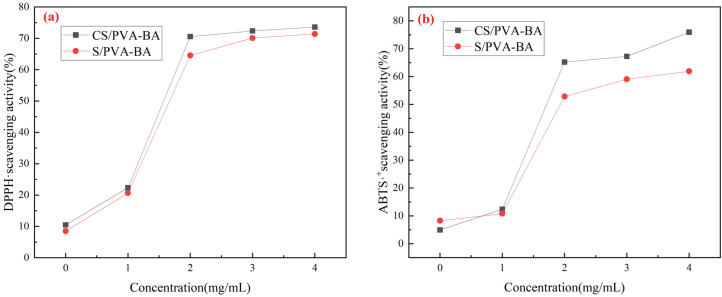
Antioxidant properties of the gels. (**a**) DPPH radical scavenging activity of the gels; (**b**) ABTS radical scavenging activity of the gels.

The antioxidant properties of the gels were evaluated through DPPH and ABTS radical scavenging assays, as shown in Figure 4. The DPPH and ABTS radical scavenging rates of the CS/PVA gel were 11.69% and 5.98%, respectively, while those of the S/PVA gel were 8.28% and 8.97%. The antioxidant activity of the gels without anthocyanins was relatively low. However, with increasing anthocyanin concentrations, the scavenging rates of CS/PVA (4 mg/mL) reached 73.59% and 77.47%, and those of S/PVA (4 mg/mL) reached 71.69% and 62.46%, demonstrating significant antioxidant capacity. This activity is attributed to the phenolic hydroxyl groups (−OH) in anthocyanins, which donate hydrogen atoms or electrons to neutralize reactive oxygen species (ROS) such as superoxide anions (O_2_^−^), hydroxyl radicals (·OH), and hydrogen peroxide (H_2_O_2_), thereby interrupting free radical chain reactions and delaying lipid oxidation and protein denaturation [43]. Notably, the antioxidant efficacy of gels depends on the interaction between active components and the polymer matrix [44]. Factors such as the cross-linking status, moisture content, temperature, and microstructure of the gels may also influence the accessibility and stability of the antioxidant compounds [45].

### 2.4. Analysis of Sensitivity of Complex Polysaccharide Gels to Ammonia

**Figure 5:**
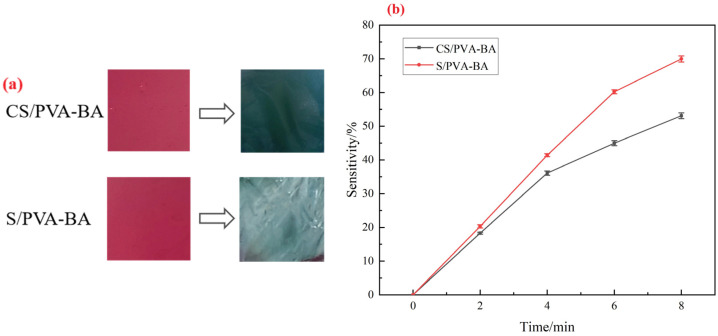
Response of the gels to ammonia. (**a**) Color change in the gels after 8 min of ammonia exposure. (**b**) Sensitivity of gel response to ammonia. Vertical bars represent the standard deviations of five replicates.

Volatile nitrogen-containing compounds such as ammonia, trimethylamine, and dimethylamine will be produced in the spoilage process of high-protein foods, resulting in an increase in the pH value in food packaging [46]. Simulating this process with ammonia gas is used to assess the sensitivity of the complex polysaccharide gels to volatile alkaline gases. The release of volatile amines causes the pH in the food packaging environment to rise, and anthocyanins are sensitive to pH changes, resulting in the gel in use showing a significant color change [28]. Figure 5a shows the color changes in the two gels in an ammonia environment in 8 min, and Figure 5b demonstrates the response kinetics and sensitivity of two indicator gels to ammonia. The rate of color change in the gels varied over time. During the initial 4 min, both CS/PVA-BA and S/PVA-BA gels exhibited rapid sensitivity enhancement, indicating their high reactivity and fast response to ammonia. Notably, S/PVA-BA achieved a maximum sensitivity of 69.931% at 8 min while CS/PVA-BA also showed significant responsiveness with a sensitivity of 53.016% at the same time point. The sensitivity enhancement during the reaction primarily resulted from NH_3_ molecules interacting with water in the gel matrix, generating an alkaline environment that increased the gel’s swelling capacity and thereby amplified its chromatic response.

### 2.5. Application of Complex Polysaccharide Gels in Beef Freshness Monitoring

#### 2.5.1. Analysis of Changes in Beef Physicochemical Properties

**Figure 6:**
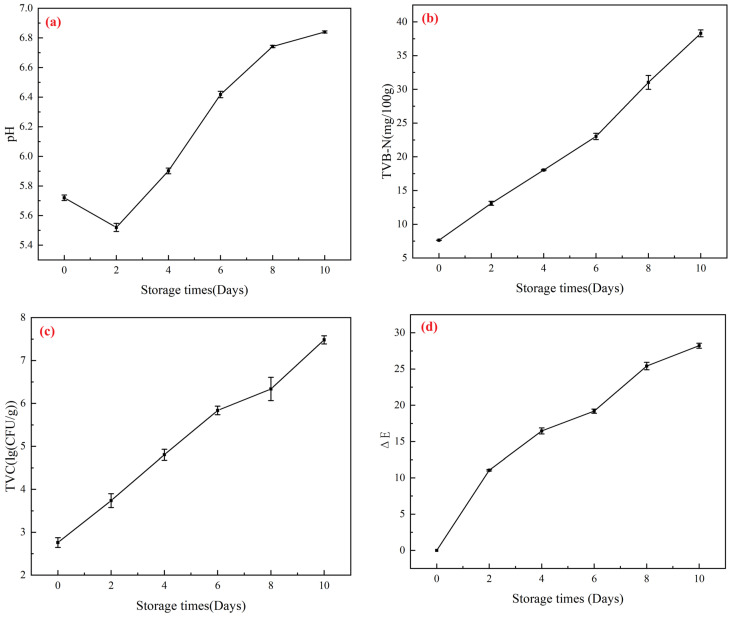
Application of the gels in beef freshness monitoring. (**a**) Changes in pH value during beef storage. (**b**) Changes in TVB-N value during beef storage. (**c**) Changes in the total number of colonies during beef storage. (**d**) Changes in the values during beef storage. Vertical bars represent the standard deviations of five replicates.

The spoilage of beef during storage at 4 °C was reflected by changes in total volatile basic nitrogen (TVB-N), which indicates protein degradation and microbial metabolic activity. According to national standards, fresh meat should have a TVB-N value ≤ 15 mg/100 g [47]. Beef is classified as sub-fresh when TVB-N ranges between 15 and 25 mg/100 g and spoiled when exceeding 25 mg/100 g [48]. As shown in the Figure 6, the initial TVB-N value of beef was 7.65 mg/100 g. After 2 days, it increased to 13.25 mg/100 g, approaching the upper limit for fresh meat. By day 8, the TVB-N value reached 31.25 mg/100 g, indicating complete spoilage with extensive protein breakdown and the production of hydrogen sulfide (H_2_S) and amines [49].

The pH evolution of chilled beef is shown in Figure 6a. Freshness criteria classify meat as follows: pH 5.8–6.2 (fresh), pH 6.3–6.6 (sub-fresh), and pH > 6.7 (spoiled) [50]. The initial pH of fresh beef was 5.72. The post-slaughter glycolysis of residual glycogen under anaerobic conditions produced lactic acid, temporarily lowering the pH to 5.52 by day 2 [51]. During this phase, microbial activity (e.g., lactic acid bacteria) further decomposed carbohydrates, generating organic acids (e.g., lactic acid and acetic acid) and slightly reducing the pH [52]. Subsequently, dominant spoilage bacteria (e.g., Pseudomonas) metabolized proteins and amino acids, releasing alkaline metabolites (e.g., ammonia and amines), leading to a significant pH increase to 6.75 by day 8, confirming spoilage.

Microbial growth and metabolism are the primary drivers of meat spoilage [53]. Freshness standards define total viable counts (TVCs) as follows: <4.0 lg (CFU/g) (fresh), 4.0–6.0 lg (CFU/g) (sub-fresh), and >6.0 lg (CFU/g) (spoiled) [54]. A TVC exceeding 7.0 lg (CFU/g) indicates complete deterioration. As shown in Figure 6c, the initial TVC of beef was 2.67 lg (CFU/g). By day 4, it increased to 4.87 lg (CFU/g), categorizing the beef as sub-fresh. After 6 days, the TVC surpassed 6.0 lg (CFU/g), marking the onset of spoilage.

#### 2.5.2. Freshness Monitoring Experiment

**Figure 7:**
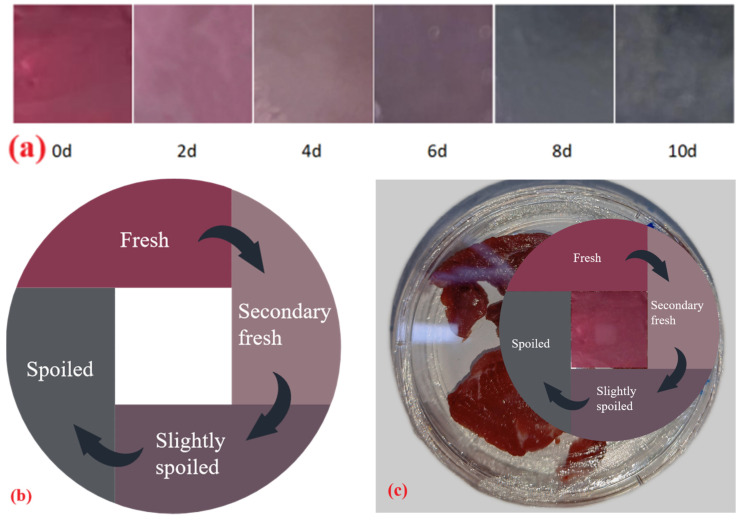
The color changes in the CS/PVA-BA gel for monitoring the freshness of beef stored at 4 °C. (**a**) Color changes in the gels at different storage times. (**b**) Label card image. (**c**) Usage of the label card.

The CS/PVA-BA gel, selected for its high sensitivity and superior performance, was applied to monitor the freshness of chilled beef. Figure 7a illustrates the chromatic transitions of the gel during the 10-day storage of beef at 4 °C. Initially, the gel exhibited a magenta-red hue, which transitioned to pale magenta-red as storage progressed. Concurrent with the continuous accumulation of volatile nitrogenous compounds, the gel darkened and shifted toward bluish tones, turning bluish-gray by day 8 and fading to light bluish-gray by day 10. These color variations were visually discernible throughout the storage period.

Figure 6d, corresponding to Figure 7a, presents the color difference (ΔE) values of the gel during refrigeration. Higher ΔE values indicate more pronounced chromatic shifts. The CS/PVA-BA gel demonstrated a rapid increase in ΔE, reaching 16.51 by day 4 (visually noticeable color change) and 27.93 by day 8 (bluish-gray appearance). The gel’s chromatic evolution closely correlated with beef quality deterioration, validating its applicability in detecting freshness.

A colorimetric indicator label (Figure 7b) was developed based on these transitions, enabling consumers to assess real-time beef freshness through visual comparison. The label features referenced colors corresponding to storage days 0, 4, 6, and 8. Day 0 (magenta-red): Fresh and safe for consumption. Day 4 (pale pink): Sub-fresh; immediate use recommended. Day 6 (pale magenta-red): Early spoilage. Day 8 (bluish-gray): Fully spoiled. This system will allow consumers to visually determine beef freshness, achieving the real-time monitoring of product quality.

## 3. Conclusions

Anthocyanins extracted from blueberry pomace were utilized as pH indicators to develop freshness-indicating gels by combining them with film-forming matrices of starch/polyvinyl alcohol (S/PVA) and chitosan/polyvinyl alcohol (CS/PVA). Four types of gels were prepared based on the presence or absence of anthocyanins. Microstructural analysis revealed good compatibility between the matrices and anthocyanins, with anthocyanin incorporation improving the physicochemical properties of the gels. Mechanical and sensitivity evaluations demonstrated that the CS/PVA-BA gel exhibited the highest elongation at break, a low water content (8.33 ± 0.57%), and reduced water vapor permeability (WVP) while the S/PVA-BA gel showed superior tensile strength and higher pH-responsive sensitivity. DPPH and ABTS radical scavenging assays confirmed the antioxidant capacity of the gels, effectively extending the shelf life of chilled beef. The CS/PVA-BA gel, selected for its optimal performance, was applied to monitor the freshness of chilled beef. Physicochemical analysis indicated initial spoilage at 48 h (TVB-N > 15 mg/100 g, TVC > 4.0 log CFU/g), with the gel transitioning from magenta-red to pale magenta-red. By day 6 of refrigeration, advanced spoilage was marked by a bluish-gray color shift. A smart colorimetric label card was developed to visually indicate freshness stages (0–8 days), enabling consumers to assess beef quality in real time. This composite polysaccharide gel integrates freshness monitoring and preservation functionalities, demonstrating significant potential for intelligent meat packaging. The study has provided guidance for the high-value utilization of blueberry pomace and aligned with current trends in food safety, smart packaging, and sustainable development.

## 4. Materials and Methods

### 4.1. Materials and Reagents

Blueberry pomace was provided by the Horticulture Branch of Heilongjiang Academy of Agricultural Sciences (2022); chilled fresh beef was purchased from a local supermarket (Harbin, China); polyvinyl alcohol (PVA) (degree of polymerization = 1700–1800; 88% alcoholysis), chitosan (CS) (deacetylated grade 85%), and starch (S) were obtained from Shanghai Macklin Biochemical Technology Co., Ltd., Shanghai, China; glycerol and citric acid were sourced from Shanghai Pinchen Biotechnology Co., Ltd., Shanghai, China. All other chemicals were analytical-grade and were purchased from Shanghai Macklin Biochemical Technology Co., Ltd., Shanghai, China.

### 4.2. Extraction and Quantification of Anthocyanins from Blueberry Pomace

Anthocyanins were extracted following the method of Fan [55] with slight modifications. Briefly, 200 g of frozen blueberry pomace was weighed using an electronic balance, mixed with 400 mL of 60% (*v*/*v*) acidified ethanol (pH around 4.5), and soaked at room temperature for 2.5 h. The mixture was homogenized for 5 min in a tissue homogenizer (WBL80Y21, Shanghai Yuanmai Trading Co., Ltd., Shanghai, China) and the homogenate was subjected to vacuum filtration. The filtrate was concentrated to near dryness under reduced pressure at 42 °C using a rotary evaporator (Rotavapor R-300, Buchi Labortechnik AG, Flaville, Switzerland). The concentrate was then adsorbed onto AB-8 macroporous resin, eluted with ethanol, and dried at 40 °C for 24 h to obtain anthocyanins.

The total anthocyanin content (TAC) following the method of Merz [21] in the extract was determined by spectrophotometer (UV-1800, Shimadzu Corporation, Kansai, Japan). Equal aliquots of 0.5 mL sample (1 mg/mL) were dissolved in 4.5 mL of 0.025 M potassium chloride buffer (pH = 1) and 4.5 mL of 0.4 m sodium acetate buffer (pH = 4.5) and placed in different test tubes. The absorbance of the extract was determined at 525 nm (A525 nm) and 700 nm (A700 nm). TAC was calculated using Equation (1):

(1) $$TAC(mg/g)=\frac{\left[{\left(A_{525nm}-A_{700nm}\right)}_{pH=1.0}-{\left(A_{525nm}-A_{700nm}\right)}_{pH=4.5}\right]M\omega DF}{\varepsilon L}$$

Here, M, DF, ε, and L were the molecular weight of anthocyanin-3-glycoside (449.2 g/mol), dilution factor (10), molar extinction coefficient (29,600 L/(cm, mol)), optical path length (1 cm), and blank, respectively.

### 4.3. pH Response of Anthocyanin Solution

#### 4.3.1. Color Response of Anthocyanins to pH

Buffer solutions (pH 2–14) were prepared and anthocyanin solution was added to each at a final concentration of 0.01% (*w*/*v*). Color changes were observed and photographed. The L*, a*, and b* values of the solution were determined by a colorimeter (CR-400/410, Konica Minolta, Inc., Tokyo, Japan). Total color difference (ΔE) was calculated using Equation (2):

(2) $$\Delta E=\sqrt{{\left(L^{*}-L_{0}\right)}^{2}+{\left(a^{*}-a_{0}\right)}^{2}+{\left(b^{*}-b_{0}\right)}^{2}}$$

Here, L*= the measured brightness value of the sample, a* = the measured red–green value of the sample, b* = the measured yellow–blue value of the sample; L_0_ = the initial brightness value of the sample, a_0_ = the initial red–green value of the sample, and b_0_ = the initial yellow–blue value of the sample.

#### 4.3.2. UV-VIS Spectra of Anthocyanins at Different pH Values

UV-VIS spectra of anthocyanin solutions (pH 2–14) were recorded using a spectrophotometer (UV-1800, Shimadzu Corporation, Japan) at wavelengths of 400–800 nm.

### 4.4. Preparation of Complex Polysaccharide Gels

Gels were prepared based on Xue’s method with modifications [56]. CS, S, and PVA solutions were mixed to form gel substrates. CS solution was prepared by dissolving 1 g of chitosan in 100 mL of citric acid solution under magnetic stirring at 20 °C for 30 min. S solution (1 g starch/100 g solution) and PVA solution (1 g PVA/100 g solution) were prepared by heating at 95 ± 1 °C and 80 ± 1 °C, respectively.

Solutions were mixed at CS/PVA (60/40, %*v*/*v*) and S/PVA (40/60, %*v*/*v*) ratios. CS/PVA gel was prepared from a suspension of chitosan (0.6, %*m*/*m*), polyvinyl alcohol (0.4, %*m*/*m*), citric acid solution (0.25, %*m*/*m*), and glycerol (1, %*m/m*); S/PVA gel was prepared from a suspension of starch (0.4, %*m*/*m*), polyvinyl alcohol (0.6, %*m*/*m*), citric acid solution (0.25, %*m*/*m*), and glycerol (1, %*m*/*m*), followed by stirring at 20 °C for 30 min [57]. Gels were cast by pouring 18 mL of degassed solution into 9 cm Petri dishes and dried at 36 °C. The gel was colorless and transparent. The resulting gels (CS/PVA, S/PVA) were stored in the dark. The solution was mixed in a CS/PVA ratio of 60/40 (%*v*/*v*) and a S/PVA ratio of 40/60 (%*v*/*v*). CS/PVA-BA gel was prepared from a suspension of chitosan (0.6, %*m*/*m*) and polyvinyl alcohol (0.4, %*m*/*m*); S/PVA-BA gel was prepared from a suspension of starch (0.4, %*m*/*m*) and polyvinyl alcohol (0.6, %*m*/*m*). Citric acid solution (0.25, %*m*/*m*) and glycerol (1, %*m*/*m*) were added to the mixed solution, and the addition amount of blueberry pomace anthocyanin (pH = 4.5) was 2%. The mixture was stirred at 20 °C with a magnetic stirrer for 30 min. They were named CS/PVA-BA membrane and S/PVA-BA membrane, respectively. The gel was translucent and purplish-red.

### 4.5. Characterization of Gels

#### 4.5.1. Microstructural Analysis

Gel surfaces were observed using scanning electron microscopy (SEM, 5 kV acceleration voltage, 1000× magnification) (SU1510, Hitachi, Ltd., Tokyo, Japan). Samples were cryo-fractured in liquid nitrogen and sputter-coated with gold.

#### 4.5.2. FT-IR Analysis

Fourier-transform infrared (FT-IR) spectra (Nicolet iS10, Thermo Fisher Scientific Inc., Waltham, MA, USA) were recorded in the range of 4000–500 cm^−1^ with 16 scans per sample [48].

#### 4.5.3. Thickness and Mechanical Properties

Gel thickness was measured using a digital micrometer (average of 8 random points, µm) (CH-1-ST, Shanghai Precision Instrument Co., Ltd., Shanghai, China). Tensile strength (TS) and elongation at break (EB) were tested using a microcomputer-controlled electronic universal testing machine (UTM5205X, Shenzhen Suns Technology Stock Co., Ltd., Shenzhen, China) in accordance with GB/T 1040.3-2006 [58]. Samples (60 mm × 20 mm) were stretched at 36 mm/min with a 40 mm initial grip distance. TS and EB were calculated using Equations (3) and (4):

(3) $$\mathrm{TS}\left(\mathrm{Mpa}\right)=\frac{F}{\omega d}$$

Here, F = maximum load (N), ω = width (mm), and d = thickness (mm).

(4) $$\mathrm{EB}(\%)=\frac{L_{0\;}-\;L_{1}}{L_{0}}\times 100$$

Here, L_1_= final length at break (mm) and L_0_ = initial grip distance (mm).

#### 4.5.4. Moisture Content

Taking Valencia’s method and tweaking it slightly [59], samples (m_0_) were dried at 105 °C to constant weight (m_1_). Moisture content (MC) was calculated using Equation (5):

(5) $$\mathrm{MC}\left(\%\right)=\frac{m_{0}\;-{\;m}_{1}}{m_{0}}\times 100$$

Here, m_0_ = indicates the initial mass(g) of the gel and m_1_ = indicates the mass (g) of the gel after drying to a constant mass.

#### 4.5.5. Determination of Water Vapor Transmission Coefficient

Taking Zou’s method and tweaking it slightly [41], the composite gels were submerged in beakers containing 20 mL of distilled water and placed in a desiccator at 22 °C. Measurements were taken every 2 h, averaging the six weights from three parallel samples. The water vapor transmission coefficient (WVP) was calculated using Equation (6):

(6) $$\mathrm{WVP}(10^{-4}/(g\cdot \mathrm{mm})/(m^{2}\cdot h\cdot \mathrm{Pa}))=\frac{\Delta m\;\times \;x}{A\;\times \;\Delta p\;\times \;t}$$

Here, Δm = the mass of water transmitted (g), x = the thickness of the gel (mm), A = the effective permeable area of the gel (m^2^), Δp = the pressure difference between the two sides of the membrane (3179 Pa (22 °C)), and t = the interval time (s).

### 4.6. Antioxidant Properties

#### 4.6.1. DPPH Radical Scavenging Activity

Gels (0.1 g) were soaked in 10 mL distilled water for 24 h. The extract (1 mL) was mixed with 3 mL of 0.1 mmol/L DPPH-methanol and incubated in the dark for 30 min. Absorbance was measured at 517 nm through spectrophotometry (UV-1800, Shimadzu Corporation, Kansai, Japan). DPPH scavenging rate was calculated using Equation (7) [60]:

(7) $$\mathrm{DPPH}\left(\%\right)=\left[1-\frac{A_{X}-A_{X0}}{A_{0}}\right]\times 100$$

Here, A_X_, A_X0_, andA_0_ = absorbance of sample, control, and blank, respectively.

#### 4.6.2. ABTS Radical Scavenging Activity

ABTS working solution (absorbance 0.7 ± 0.02 at 734 nm) was prepared by mixing 7.4 mmol/L ABTS and 2.6 mmol/L potassium persulfate (1:1), followed by 12 h incubation. Sample solution (1 mL) was mixed with 4 mL ABTS working solution, incubated for 6 min, and absorbance (A) was measured. Absorbance was measured at 734 nm through spectrophotometry (UV-1800, Shimadzu Corporation, Kansai, Japan). Scavenging activity was calculated using Equation (8) [61]:

(8) $$\mathrm{ABTS}\left(\%\right)=\frac{A_{0}-A}{A_{0}}\times 100$$

Here, A = the light absorption value of the sample and A_0_ = the light absorption value of the blank.

### 4.7. Ammonia Sensitivity

According to the method of Kuswandi with appropriate modifications [62], in a 50 mL centrifuge tube containing 45 mL of 8 mmol/L ammonia solution, the gel (2 cm × 2 cm) was fixed within the culture medium and inverted on the bottle mouth. After 8 min, gel images were collected under room temperature conditions. Five sets of RGB values were extracted from the gel every two minutes using Photoshop 2018 color sampler, with mean values calculated to determine gel sensitivity. The calculation formula is shown in Equation (9):

(9) $$S(\%)=\frac{\left|R-R_{0}\right|+\left|G-G_{0}\right|+\left|B-B_{0}\right|}{R+G+B}\times 100$$

Here, R_0_, G_0_, and B_0_ are the initial values before this gel reaction; R, G, and B are the values after the reaction.

### 4.8. Application in Beef Freshness Monitoring

#### 4.8.1. Color Change

Gels were fixed on Petri dish lids and stored with beef at 4 °C. Color parameters (L*, a*, b*) were measured every 2 days using a colorimeter (CR-400/410, Konica Minolta, Inc., Tokyo, Japan). Total color difference (ΔE) was calculated using Equation (10):

(10) $$\Delta E=\sqrt{{\left(L^{*}-L_{0}\right)}^{2}+{\left(a^{*}-a_{0}\right)}^{2}+{\left(b^{*}-b_{0}\right)}^{2}}$$

Here, L* = the measured brightness value of the sample, a* = the measured red–green value of the sample, b* = the measured yellow–blue value of the sample; L_0_ = the initial brightness value of the sample, a_0_ = the initial red–green value of the sample, and b_0_ = the initial yellow–blue value of the sample.

#### 4.8.2. TVB-N Determination

Total volatile base nitrogen (TVB-N) was measured according to GB 5009.228-2016 [63]. Sample Preparation: A quantity of 10 g of beef was added to 50 mL of water. The beef sample was pre-homogenized into a fine paste, thoroughly mixed, and oscillated for 30 min. The mixture was then filtered and the filtrate was transferred to a conical flask for subsequent analysis. Determination Procedure: A condenser was inserted into a receiving flask containing 10 mL of 2% boric acid absorption solution, which was supplemented with a mixed indicator solution (0.2% methyl red ethanol solution and 0.1% methylene blue aqueous solution, mixed in a 1:1 ratio). Quantities of 5 mL of the filtered sample solution and 5 mL of a 1% magnesium oxide suspension were combined and introduced into the reaction chamber of the distillation apparatus. The chamber was sealed and distilled water was added. Steam distillation was initiated, with timing starting upon collection of the first condensate droplet. The distillation was terminated after 5 min. The absorption solution was titrated with 0.01 mol/L hydrochloric acid (HCl) until the endpoint (transition to a bluish-purple hue) was reached. A blank experiment was performed in parallel. Total volatile base nitrogen (TVB-N) was calculated using Equation (11):

(11) $$X(\mathrm{mg}/100g)=\frac{\left(V_{1}-V_{2}\right)\times c\times 14}{m}\times 100$$

Here, V1 = the volume of hydrochloric acid consumed by the sample (mL), V2 = the volume of hydrochloric acid consumed by the blank (mL), c = the concentration of hydrochloric acid (mol/L), and m = the mass of the sample (g).

#### 4.8.3. pH Measurement

Beef (10 g) was homogenized with 50 mL distilled water. The pH of the supernatant was measured after 30 min.

#### 4.8.4. Total Bacterial Count

Total viable count (TVC) was determined using GB 4789.2-2016 [64] (plate count method). Under aseptic conditions, 10 g of ground beef sample was placed into a sterile bag, mixed with 90 mL of sterile saline solution, and homogenized for 120 s. The homogenate was then serially diluted at a 1:10 ratio. Three appropriate dilution gradients were selected, and three plates were prepared for each dilution, along with blank controls. The spread plate method was performed using plate count agar (PCA). The plates were incubated at 37 °C in a constant-temperature incubator for 48 h. The total viable count was calculated, and the results were expressed in lg CFU/g (logarithmic colony-forming units per gram). Three replicates were performed for each sample.

### 4.9. Statistical Analysis

Data were analyzed using SPSS26 and Origin2021 software. Results are expressed as means ± standard deviations. ANOVA was performed, and (*p* < 0.05) indicated statistical significance.

## Figures and Tables

**Table 1:** Gel thickness, mechanical properties, moisture content, and water vapor transmission coefficient data.

Complex Polysaccharide Gels Name	Thickness/μm	Tensile Strength/MPa	Elongation at Break/%	Moisture Content/%	Water Vapor Transmission Coefficient × 10^−4^/(g·mm)/(m^2^·h·Pa)
CS/PVA	55.00 ± 2.00 ^c^	23.94 ± 1.08 ^c^	62.27 ± 1.26 ^b^	10.89 ± 0.23 ^b^	2.56 ± 0.12 ^b^
S/PVA	71.33 ± 1.51 ^b^	27.95 ± 1.69 ^a^	43.45 ± 1.09 ^c^	11.97 ± 0.57 ^a^	3.25 ± 0.31 ^ab^
CS/PVA-BA	84.48 ± 1.83 ^b^	25.42 ± 2.01 ^b^	87.84 ± 2.34 ^a^	8.33 ± 0.57 ^c^	2.68 ± 0.11 ^b^
S/PVA-BA	99.00 ± 1.55 ^a^	28.31 ± 1.28 ^a^	64.52 ± 2.33 ^b^	10.12 ± 0.15 ^b^	3.81 ± 0.21 ^a^

Values are expressed as means ± standard deviations. Different letters in the same column indicate significant differences (*p* < 0.05).

## Data Availability

All data are available within the manuscript.
